# Wisteria floribunda agglutinin-positive human Mac-2 binding protein predicts liver cancer development in chronic hepatitis B patients under antiviral treatment

**DOI:** 10.18632/oncotarget.17670

**Published:** 2017-05-07

**Authors:** Ka-Shing Cheung, Wai-Kay Seto, Danny Ka-Ho Wong, Lung-Yi Mak, Ching-Lung Lai, Man-Fung Yuen

**Affiliations:** ^1^ Department of Medicine, The University of Hong Kong, Queen Mary Hospital, Pokfulam, Hong Kong; ^2^ State Key Laboratory for Liver Research, The University of Hong Kong, Pokfulam, Hong Kong

**Keywords:** WFA^+^-M2BP, NA therapy, HCC, HBV, cirrhosis

## Abstract

**AIM:**

The risk factors for hepatocellular carcinoma (HCC) development in chronic hepatitis B (CHB) patients with undetectable serum HBV DNA under nucleos(t)ide analogue (NA) therapy are not well defined. We aimed to examine the relationship between Wisteria floribunda agglutinin-positive human Mac-2 binding protein (WFA^+^-M2BP) and HCC development in these patients.

**Results:**

There was a significant difference in the median levels of pre-treatment WFA+-M2BP between the HCC and control groups (0.67 vs 0.41 COI, respectively, *p* < 0.001). Among patients with cirrhosis, the median level of WFA^+^-M2BP was higher in HCC group than in control group (0.74 vs 0.47 COI, respectively, *p* = 0.014). Among patients without cirrhosis, the median level of WFA^+^-M2BP of HCC group was also higher (0.48 vs 0.28 COI, respectively, *p* = 0.002). With a cutoff value of 0.69, the AUROC of pre-treatment WFA^+^-M2BP to predict HCC development for the whole cohort was 0.70. With cutoff values of 0.69 and 0.34, the AUROCs to predict HCC were 0.67 and 0.77 for patients with and without cirrhosis, respectively.

**Materials and Methods:**

Fifty-seven NA-treated patients with undetectable HBV DNA who developed HCC were compared with 57 controls (matched with demographics and treatment duration). WFA^+^-M2BP levels were measured, and expressed as cutoff index (COI). Subgroup analyses were also performed in patients with and without cirrhosis.

**Conclusions:**

A higher pre-treatment WFA+-M2BP level was associated with an increased risk of HCC development in patients with undetectable HBV DNA under NA therapy. Further longitudinal studies are required to examine the role of WFA+-M2BP as an accessory risk marker for HCC development.

## INTRODUCTION

Hepatitis B virus (HBV) infection is one of the commonest chronic infections, affecting 248 million people worldwide [1], with more than 1 million people dying from HBV-related diseases annually [2]. Chronic hepatitis B (CHB) infection can lead to various adverse outcomes, including hepatitis flares, cirrhosis and hepatocellular carcinoma (HCC) [3]. Among various risk factors for HCC development, cirrhosis and high serum HBV DNA levels (≥ 2,000 IU/mL) are the major ones [3, 4].

Chronic hepatitis results in regeneration and cirrhosis. The risk of HCC is significantly increased in case of cirrhosis, due to the activation of oncogenes, overexpression of growth factors and inactivation of tumor suppressor genes [5]. Therefore, it is of paramount importance in assessing the severity of liver fibrosis and cirrhosis, in an accurate and timely manner. This helps in prognostication and in turns affects management like antiviral treatment initiation, endoscopic screening for varices and screening for HCC. Although liver biopsy is a gold standard, it is an invasive procedure associated with various complications which occasionally can be life-threatening [6]. Currently available non-invasive methods include direct and indirect serum markers, transient elastography, and magnetic resonance (MR) elastography. However, each of these investigatory modalities is associated with certain disadvantages [7]. Recently, a lectin-antibody sandwich immunoassay has been developed for measuring a novel serum glyco-marker for assessing liver fibrosis. This serum marker is known as the Wisteria floribunda agglutinin positive Mac-2 binding protein (WFA^+^-M2BP) [8, 9]. The test has the advantages of being simple, rapid and noninvasive.

M2BP has a special characteristic that changes its sugar chain structure following the progression of hepatic fibrosis. WFA is a lectin used to recognize the altered glycan parts of the M2BP secreted from the diseased cells [8]. WFA^+^-M2BP has been proven to be a useful marker in quantification of fibrosis in patients with hepatitis [8, 10]. The diagnostic performance of this test is superior to many currently available surrogate markers of fibrosis including APRI (AST to platelet ratio index), hyaluronic acid and type IV collagen peptide [10]. Clinical applicability of this marker in fibrosis has been studied in patients with viral hepatitis [11–14].

Apart from determining the degree of fibrosis, WFA^+^-M2BP has been found to be a useful surrogate marker for predicting HCC development in hepatitis C virus (HCV) patients [15, 16]. Recently, a few retrospective studies also demonstrated the potential role of this marker in predicting development of HCC in CHB patients [11, 12, 17]. However, these studies involved a heterogeneous group of patients who were both treatment-naïve and treatment-experienced (with either interferon or NA therapy). Although several existing models can predict HCC development in treatment-naïve patients, there are no satisfactory tests to predict HCC in patients under treatment [18]. With potent nucleos(t)ide analogues (entecavir and tenofovir), majority of CHB patients are able to achieve profound viral suppression with undetectable serum HBV DNA level. However, antiviral therapy can only reduce but not eliminate the risk of HCC [19].

Therefore, our study aimed to investigate the association of WFA^+^-M2BP with HCC development in patients with undetectable HBV DNA by NA therapy.

## RESULTS

### Patient characteristics

The demographics of the study population (57 HCC patients and 57 controls) are illustrated in Table 1. There were no significant differences between the two groups in terms of age, gender, HBeAg status, cirrhosis status, serum HBV DNA level and duration of therapy. The median age of the HCC group was similar to that of the control group (61.4 and 61.1 years, respectively, *p* = 0.764). Eighty-four percent of the cohort were male, 98.2% were HBeAg-negative, and 59.6% had cirrhosis. The median duration of NA therapy was also comparable between the two groups (3.2 and 3.6 years, respectively, *p* = 0.626). There were no significant differences in other variables, except for a higher median AFP (11 vs 3 ng/mL, *p <* 0.001) and ALT level (29 vs 23 U/L, *p* = 0.009) in the HCC group.

**Table 1:** Demographics of 114 CHB patients

	HCC (*n* = 57)	Non-HCC (*n* = 57)	*p* value
**Age^#^, years**	61.4 (55.9–62.0)	61.1 (56.9–66.3)	0.764
**Male^#^, *n* (%)**	48 (84.2%)	48 (84.2%)	n.a.
**HBeAg-negative^#^, *n* (%)**	56 (98.2%)	56 (98.2%)	n.a.
**Cirrhosis^#^, *n* (%)**	34 (59.6%)	34 (59.6%)	n.a.
**Albumin, g/L**	44 (41–46)	44 (42–46)	0.813
**Bilirubin, umol/L**	12 (8–15)	11 (8–17)	0.370
**ALT, U/L**	29 (20–38)	23 (20–28)	0.009
**Platelet, x 10 ^9/L**	163 (115–203)	168 (137–215)	0.151
**INR**	1.1 (1–1.1)	1.1 (1–1.1)	0.865
**AFP, ng/mL**	11 (5–32)	3 (2–4)	< 0.001
**Undetectable DNA#, *n* (%)**	57 (100%)	57 (100%)	n.a.
**Duration of therapy#, years**	3.2 (2.0–5.0)	3.6 (2.2–5.1)	0.626

Continuous variables expressed as median (interquartile range).

CHB, chronic hepatitis B virus; HCC, hepatocellular carcinoma; ALT, alanine aminotransferase; INR, international normalised ratio; AFP, alpha-fetal protein.

^#^Matched variables.

n.a., *p*-value not applicable as these were matched categorical variables.

At the time of commencement of NA therapy, both groups did not have a significant difference in the median ALT levels (99 U/L [IQR: 51–192] and 95 U/L [IQR: 55–153] in HCC and control groups, respectively). With regard to the fibrosis status, there was no significant difference in the proportions of patients with significant fibrosis (FIB-4 > 1.45) between the HCC and control groups, both before treatment (89.5% vs 86.0%, respectively, *p* = 0.326) and after treatment (82.5% vs 75.4%, respectively, *p* = 0.358).

Table 2 shows the demographics of patients stratified according to the status of cirrhosis. The proportions of patients receiving different NA therapies are listed in Table 3.

**Table 2:** Demographics of 114 CHB carriers stratified according to cirrhosis status

	Cirrhosis-positive (*n* = 68)			Cirrhosis-negative (*n* = 46)		
	HCC (*n* = 34)	Non-HCC (*n* = 34)	*p* value	HCC (*n* = 23)	Non-HCC (*n* = 23)	*p* value
**Age#, years**	61.1 (57.7–62.9)	62.4 (57.9–62.2)	0.797	62.0 (54.2–66.7)	60.7 (53.2–68.3)	0.974
**Male#, *n* (%)**	20 (87.0%)	20 (87.0%)	1.00	28 (82.4%)	28 (82.4%)	n.a.
**HBeAg-ve#, *n* (%)**	22 (95.7%)	22 (95.7%)	1.00	34 (100%)	34 (100%)	n.a.
**Albumin, g/L**	43 (40–47)	44 (42–46)	0.309	45 (42–46)	43 (41–46)	0.458
**Bilirubin, umol/L**	11 (7–17)	11 (9–17)	0.927	12 (9–17)	10 (8–15)	0.183
**ALT, U/L**	34 (24–43)	23 (20–29)	0.002	25 (17–32)	21 (20–28)	0.692
**Platelet, x 10 ^9/L**	132 (91–170)	153 (125–192)	0.102	188 (166–215)	208 (174–259)	0.199
**INR**	1.1 (1.0–1.1)	1.1 (1.0–1.1)	0.385	1.1 (1.0–1.1)	1.0 (1.0–1.0)	0.166
**AFP, ng/mL**	11 (5–27)	3 (2–4)	< 0.001	14 (7–53)	3 (2–3)	< 0.001
**Undetectable DNA#, *n* (%)**	34 (100%)	34 (100%)	1.00	23 (100%)	23 (100%)	n.a.
**Duration of therapy#, years**	3.4 (2.0–5.1)	3.7 (2.1–5.1)	0.652	3.2 (2.1–4.7)	3.4 (2.5–5.0)	0.582

Continuous variables expressed as median (interquartile range).

CHB, chronic hepatitis B virus; HCC, hepatocellular carcinoma; ALT, alanine aminotransferase; INR, international normalised ratio; AFP, alpha-fetal protein.

^#^ Matched variables.

n.a., *p*-value not applicable as these were matched categorical variable.

**Table 3:** Different NA therapies in HCC and control groups

	HCC< (*n* = 57)	Non-HCC (*n* = 57)
**Entecavir**	38 (66.7%)	50 (87.7%)
**Lamivudine**	9 (15.8%)	3 (5.3%)
**Telbivudine**	1 (1.8%)	1 (1.8%)
**Adefovir**	6 (10.5%)	2 (3.5%)
**Tenofovir**	3 (5.3%)	1 (1.8%)

NA, nucleos(t)ide analogue; HCC, hepatocellular carcinoma.

### Correlation between pre-treatment WFA^+^-M2BP and other variables

Pre-treatment WFA^+^-M2BP had a weak positive correlation with age (*r* = 0.21, *p* = 0.027) and pre-treatment HBV DNA level (*r* = 0.19, *p* = 0.050). A positive correlation also existed between WFA^+^-M2BP and FIB-4 (*r* = 0.63, *p <* 0.001), bilirubin (*r* = 0.43, *p* < 0.001) and INR (*r* = 0.21, *p* = 0.027), while there were negative correlations with albumin (*r* = –0.49, *p* < 0.001) and platelet (*r* = –0.32, p = 0.001). WFA^+^-M2BP did not have correlation with ALT (*r* = 0.12, *p* = 0.203) and AFP (*r* = –0.001, *p* = 0.995). These results indicate a positive relationship between WFA^+^-M2BP and adverse liver function.

### Comparison of pre- and post-treatment WFA^+^-M2BP levels between the HCC and control groups

**Figure 1:**
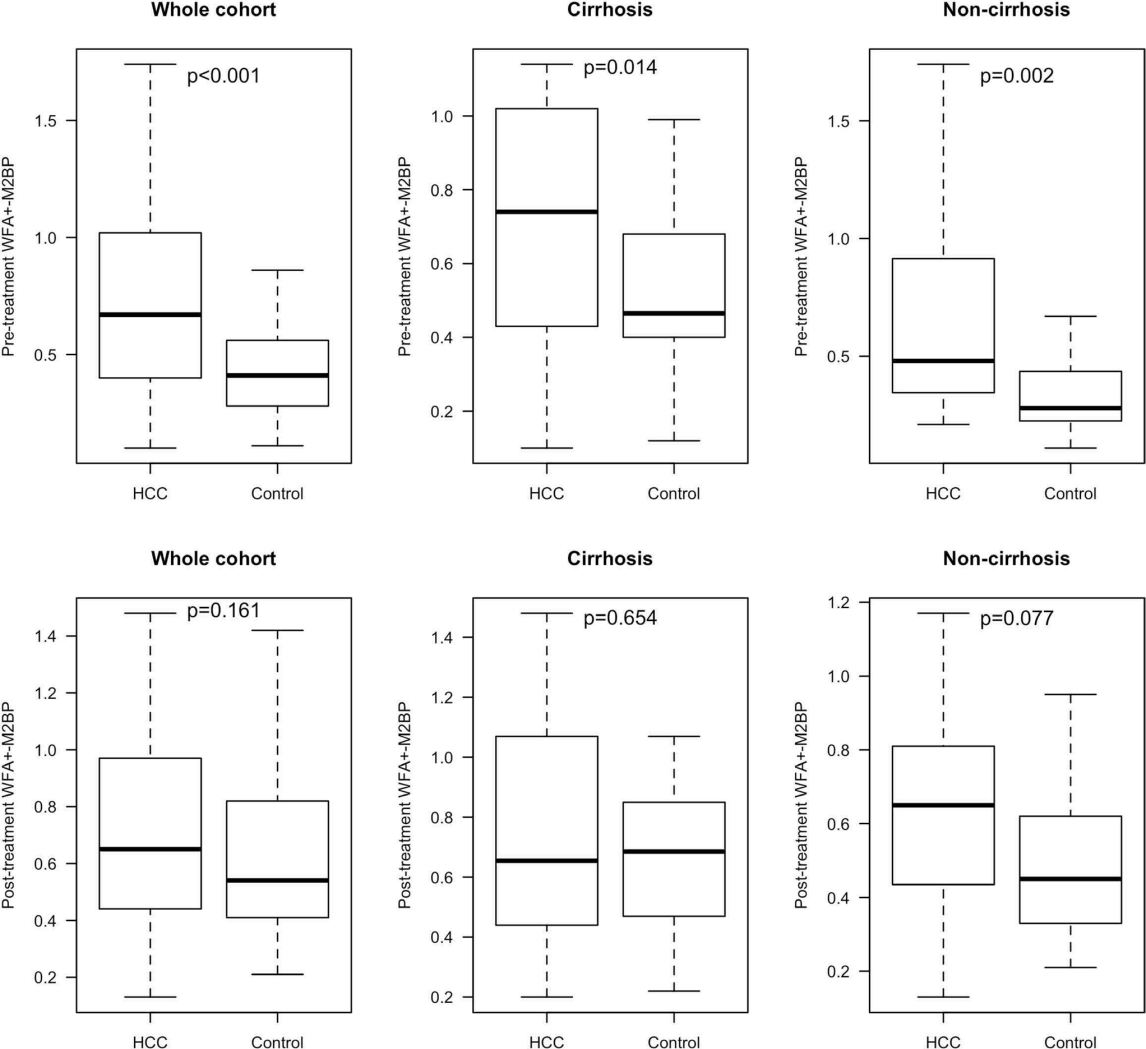
Pre-treatment and post-treatment WFA^+^-M2BP levels between the HCC and control groups Abbreviations: HCC, hepatoceullar carcinoma

Figure 1 shows the pre-treatment and post-treatment *WFA^+^-M2BP* levels between the HCC and control groups for the whole cohort and also according to cirrhosis status.

The median level of pre-treatment WFA^+^-M2BP was significantly higher in the HCC than the control groups (0.67 COI, IQR: 0.40–1.02 vs 0.41 COI, IQR: 0.28–0.56, respectively; *p* < 0.001). Among patients with cirrhosis (*n* = 68), the median level of WFA^+^-M2BP remained significantly higher in the HCC than the control groups (0.74 COI, IQR: 0.43–1.02 vs 0.47 COI, IQR: 0.40–0.68, respectively; *p* = 0.014). Among patients without cirrhosis (*n* = 46), there was also a significant difference in the median levels of WFA^+^-M2BP between the HCC and control groups (0.48 COI, IQR: 0.35–0.92 vs 0.28 COI, IQR: 0.23–0.44, respectively; *p* = 0.002).

However, there was no significant difference in the median levels of post-treatment WFA^+^-M2BP between the HCC and control groups (0.65 COI, IQR: 044–0.97 vs 0.54 COI, IQR: 0.41–0.82, respectively; *p* = 0.161). Subgroup analysis also did not reveal any significant associations (cirrhosis-positive patients: 0.66 COI, IQR: 0.45–1.05 in HCC group vs 0.69 COI, IQR: 0.47–0.85 in control group, *p* = 0.654; cirrhosis-negative patients: 0.65 COI, IQR: 0.44–0.81 in HCC group vs 0.45 COI, IQR 0.33–0.62 in control group, *p* = 0.077).

### The role of pre-treatment WFA^+^-M2BP in predicting HCC development

**Figure 2:**
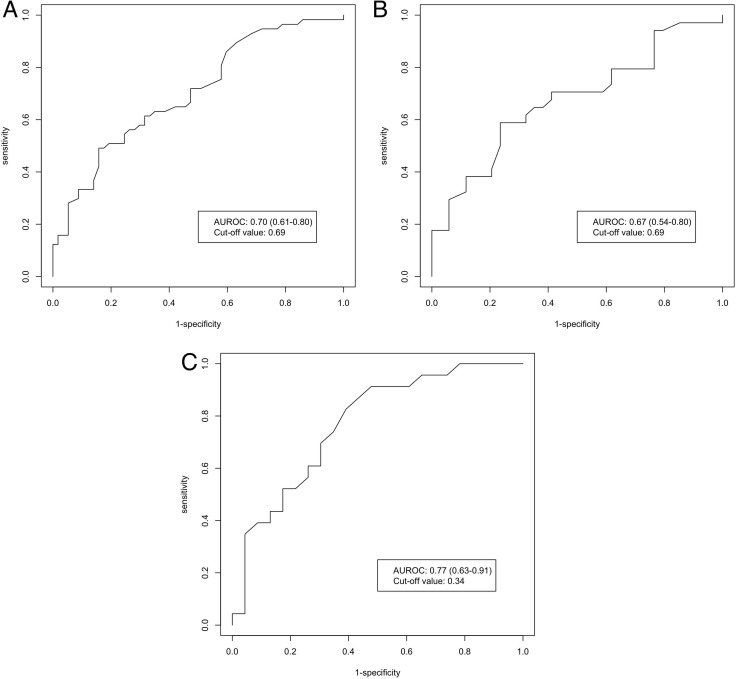
AUROC of pre-treatment WFA^+^-M2BP for HCC prediction in CHB patients (**A**) CHB patients (whole cohort); (**B**) CHB patients with cirrhosis; (**C**) CHB patients without cirrhosis; Abbreviations: AUROC, area under receiveroperating curve; WFA^+^-M2BP, Wisteria floribunda agglutinin-positive human Mac-2 binding protein; HCC, hepatocellular carcinoma; CHB, chronic hepatitis B.

By maximizing the Youden's index, the cutoff values of pre-treatment WFA^+^-M2BP to predict HCC development in CHB patients who achieved undetectable serum HBV DNA while on NA therapy were 0.69, 0.69 and 0.34 for the whole cohort, cirrhotic patients and non-cirrhotic patients, respectively. The corresponding AUROC was 0.70 (95% CI: 0.61–0.80) (Figure 2A), 0.67 (95% CI: 0.54–0.80) (Figure 2B) and 0.77 (95% CI: 0.63–0.91) (Figure 2C), respectively. Table 4 shows the predictive accuracies of pre-treatment WFA^+^-M2BP in different groups of patients. For the whole cohort, this test has a high specificity in identifying patients without HCC development (84%). Among patients without cirrhosis, with a lower cutoff value, this test has a high sensitivity in identifying patients with future HCC development (83%). In the whole study cohort, compared with patients with a pre-treatment WFA^+^-M2BP level < 0.69 COI, the OR of HCC development in patients with pre-treatment WFA^+^-M2BP ≥ 0.69 COI was 4.80 (95% CI: 1.83–12.58; *p* = 0.001).

**Table 4:** Predictive accuracies of HCC for different cutoff values of pre-treatment WFA^+^-M2BP in different groups of patients

	Whole cohort (WFA^+^-M2BP ≥ 0.69 COI)	Cirrhosis-positive (WFA^+^-M2BP ≥ 0.69 COI)	Cirrhosis-negative (WFA^+^-M2BP ≥ 0.34 COI)
**Sensitivity**	49%	59%	83%
**Specificity**	84%	76%	61%
**Positive-predictive value**	76%	71%	68%
**Negative-predictive value**	62%	65%	78%

HCC, hepatocellular carcinoma; WFA^+^-M2BP, Wisteria floribunda agglutinin positive Mac-2 binding protein.

### Comparison of pre-treatment HBV DNA levels between the HCC and control groups

There existed no significant difference in the median levels of pre-treatment HBV DNA between the HCC and control groups (6.5 and 6.0 log_10_IU/mL, respectively, *p* = 0.092). Univariate analysis in terms of categorical variable (with two cutoff values of either 2,000 IU/mL or 20,000 IU/mL) did not show any significant association between pre-treatment HBV DNA level and HCC development (*p* = 0.706 and 0.808, respectively).

### WFA^+^-M2BP levels in patients with cirrhosis

Among the controls who did not have HCC (*n* = 57), the median level of pre-treatment WFA^+^-M2BP was significantly higher in patients with cirrhosis (0.47 COI; IQR: 0.40–0.68) than that of patients without cirrhosis (0.28 COI; IQR: 0.23–0.44) (*p* = 0.014).

Similarly, there was a significant difference in the median level of post-treatment WFA^+^-M2BP between the two subgroups (cirrhosis-positive patients: 0.69 COI, IQR: 0.47–0.85 vs cirrhosis-negative patients: 0.45 COI, IQR: 0.33–0.62; *p* = 0.015).

## DISCUSSION

To date, there are a few studies examining the role of WFA^+^-M2BP in predicting the development of HCC in CHB patients [11, 12, 17]. However, these studies recruited a heterogeneous group of patients who were both treatment-naïve and treatment-experienced. For studies with treatment-experienced patients using either interferon or NA therapy, the major confounding factor, namely the DNA levels, varied.

To our knowledge, this is the first study to investigate the association between WFA^+^-M2BP and HCC development in a strictly defined cohort of patients with profound viral suppression under NA therapy. This unique group of patients is increasingly common to be encountered in daily clinical practice due to the widespread use of potent antiviral therapy, yet the risk factors for HCC development have still not been well investigated

We found that there was a significant difference in the median levels of pre-treatment WFA^+^-M2BP between HCC and control groups (0.67 and 0.41 COI, respectively), suggesting that pre-treatment WFA^+^-M2BP is one of the risk factors for HCC development in patients who are receiving NA therapy. Furthermore, on subgroup analysis, we found that this finding was consistent regardless of the cirrhosis status. This observation can be partially explained by the fact that WFA^+^-M2BP was found to correlate with the severity of fibrosis among patients without cirrhosis [11, 12, 14]. The observation that only pre-treatment but not post-treatment WFA^+^-M2BP was different between the two groups suggests that WFA^+^-M2BP is more suitable to be a predictive marker of HCC instead of a diagnostic marker.

For the whole study cohort, the OR of HCC development with pre-treatment WFA^+^-M2BP level ≥ 0.69 COI was 4.80 (95% CI: 1.83–12.58). With this cutoff value, this test has a reasonable performance with an AUROC of 0.70. Among patients without cirrhosis, with a cutoff value of 0.34, the AUROC to predict HCC was even higher (0.77) with a high sensitivity of 83%.

The findings of the present study have significant clinical implications as WFA^+^-M2BP may help to further stratify the risk of HCC development in patients with cirrhosis as well as those without cirrhosis. This in turn will help to streamline the management plan in terms of follow-up interval and HCC surveillance.

The proportion of CHB patients receiving NA therapy has been increasing. For instance, in a nationwide cohort study of Taiwan, it was found that > 40% of CHB patients were NA-experienced [20]. With potent nucleos(t)ide analogues (entecavir and tenofovir), the majority of CHB patients are able to achieve profound viral suppression with undetectable serum HBV DNA level. However, NA therapy can only reduce but not eliminate the risk of HCC [19]. As yet, there are no satisfactory tests to predict HCC in patients under treatment [18], highlighting the importance of exploring alternative predictive factors. Therefore, further follow-up studies to improve the predictive power of WFA^+^-M2BP by adding in other already identified risk factors for the development of HCC, e.g. age, gender and other viral markers, HBsAg and hepatitis B core-related antigen (HBcrAg), are highly recommended [21, 22].

Another interesting observation in the present study about the characteristics of WFA^+^-M2BP is that the levels and cut-off values tend to be lower in CHB patients than in patients with other liver diseases (including HCV, AIH, PBC, non-alcoholic fatty liver disease) [14, 15, 23–25]. In a large study investigating the role of WFA^+^-M2BP in 1,323 CHB patients (a heterogeneous group of CHB patients who were either treatment-naïve or treatment-experienced), the median WFA^+^-M2BP level was 0.61 (IQR: 0.40–1.01) [17]. While the optimal cutoff value for predicting HCC in treatment-experienced chronic HCV patients was 2.2 COI as determined by a previous study [14], the cutoff value was much lower as defined in our study (0.69 COI). This was consistent with the finding from another study on CHB patients [12], in which the optimal cutoff value for HCC prediction was 0.71 COI. In this study by Ichikawa et al, although the median levels of baseline ALT were raised (66.5 IU/L; IQR: 40–132), the median levels of baseline WFA^+^-M2BP remained to be low (0.97 COI). One postulation is that it may be related to gender variability, in which women were found to have higher WFA^+^-M2BP levels than men [15, 26]. As female accounted for more than 80% of the cohort in previous studies on AIH and PBC, while the majority of patients in our study and another study were male [12], this may partly explain the lower WFA^+^-M2BP level in our study.

There are several limitations of our study. Firstly, the role of WFA^+^-M2BP in predicting HCC is best assessed by a prospective cohort study, instead of the retrospective nature of the present study, which precludes the determination of hazard ratio of this marker. Secondly, the relatively small sample size (due to the stringent matching criteria with 6 variables) may render the study less powerful for detecting more significant differences, especially among patients with cirrhosis. Further studies will be needed to determine more precise cut-off value for the prediction of HCC development in CHB patients. However, these stringent criteria helped to minimize the selection biases inherent to a retrospective study, and to prove the association of HCC development and WFA^+^-M2BP more convincingly. Thirdly, not all patients had transient elastography or liver biopsies done, which precluded stratified analysis according to different fibrosis stages. Nonetheless, the proportions of patients with significant fibrosis (as defined by a FIB-4 value of more than 1.45) both before and after treatment were comparable between the HCC and control groups. This partly addressed the concern that the severity of fibrosis could be different between the two groups despite the matching of their cirrhosis status.

In conclusion, the present study showed that WFA^+^-M2BP could be a novel risk marker to predict HCC development in CHB patients with profound viral suppression under NA therapy. WFA^+^-M2BP measurement may serve as a useful strategy for risk stratification in terms of follow-up interval and HCC surveillance. Future prospective researches with larger sample size on the role of WFA^+^-M2BP specifically for treatment-experienced subjects are warranted.

## MATERIALS AND METHODS

### Patient recruitment

Patients were recruited from the Hepatology Clinic, Department of Medicine, The University of Hong Kong, Queen Mary Hospital, Hong Kong, from January 2007 to November 2014.

We recruited all CHB patients who developed HCC despite achieving profound viral suppression (i.e. undetectable serum HBV DNA levels by the Cobas Tagman assay) under NA therapy for at least 1 year before the diagnosis of HCC. Other inclusion criteria included age ≥ 18 years, no significant alcohol consumption (> 30 gram and > 20 gram per day for men and women, respectively), no coexisting liver diseases like HCV infection, primary biliary cholangitis (PBC), autoimmune hepatitis (AIH) and Wilson's disease, as well as no previous history of HCC.

Seventy-six HCC cases were identified. After excluding cases who did not have available serum samples for measurement of WFA^+^-M2BP level (*n* = 19), 57 patients with HCC were recruited into the present study.

**Figure 3:**
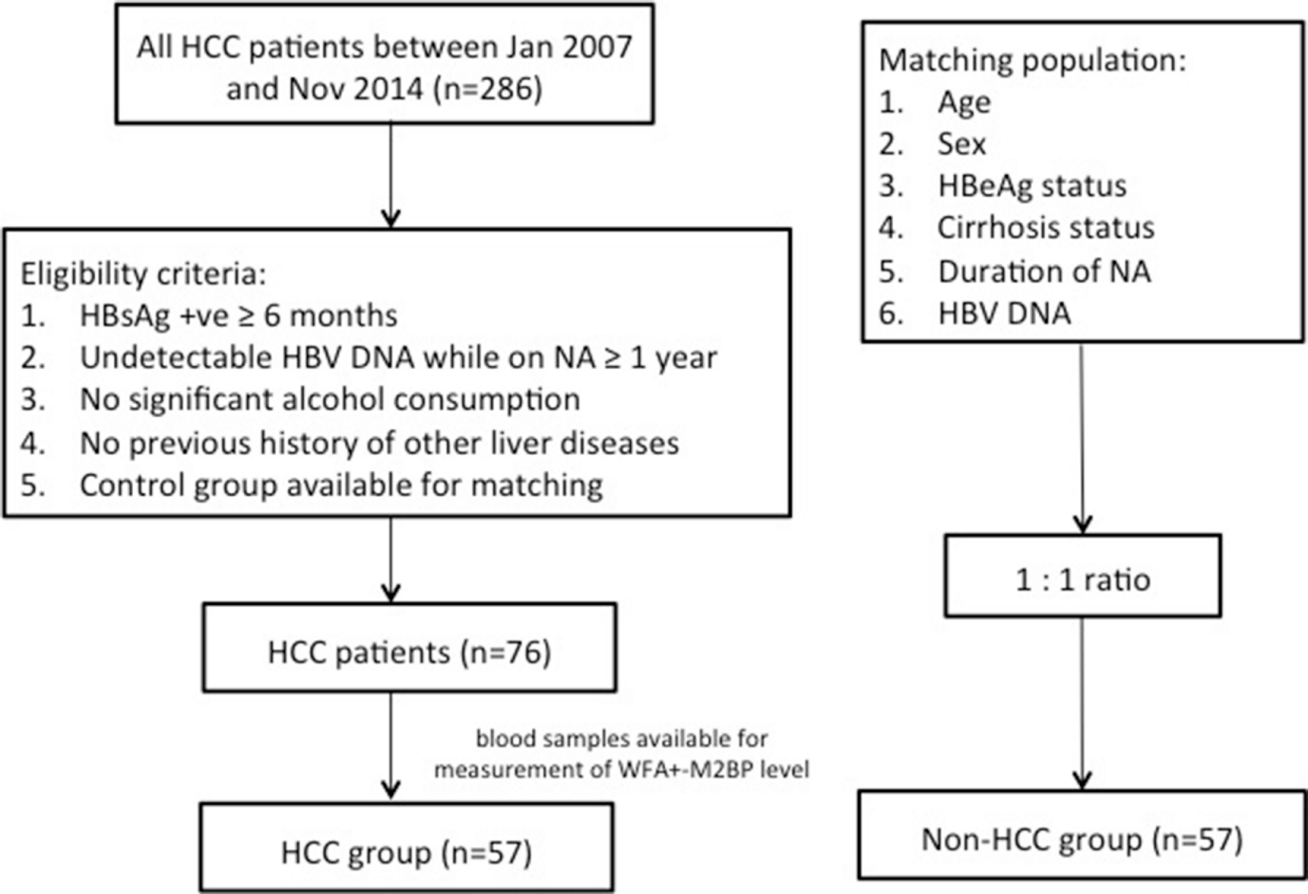
Flow chart of disposition of patients Abbreviations: HCC, hepatocellular carcinoma; HBsAg, hepatitis B surface antigen; WFA^+^-M2BP, Wisteria floribunda agglutinin-positive human Mac-2 binding protein; HBeAg, hepatitis B e antigen.

Control subjects were NA-treated CHB patients without HCC development. These control patients were matched with the HCC patients by age, gender, hepatitis e antigen (HBeAg) status, cirrhosis status (definition mentioned below) and duration of NA therapy in a 1:1 ratio. Figure 3 shows the flow diagram of the patient recruitment process.

Both pre-treatment (within 1 year before commencement of NA therapy) and post-treatment levels (at the time of HCC diagnosis for the HCC group and at the time when the control group had the same matched duration of therapy) of serum WFA^+^-M2BP were determined and compared between the two groups. Moreover, to study the association between WFA^+^-M2BP and cirrhosis, the pre-treatment and post-treatment levels of serum WFA^+^-M2BP were compared between the controls with and without cirrhosis.

The study protocol was approved by the Institutional Review Board, the University of Hong Kong and West Cluster of Hospital Authority, Hong Kong.

### Surveillance and diagnosis of HCC

We followed up the patients every 3 to 6 months with regular blood tests, which included HBeAg/anti-HBe, albumin, bilirubin, alanine aminotransferase (ALT), platelet count, international normalized ratio (INR), alpha-fetoprotein (AFP) and serum HBV DNA level. In accordance with the standard guidelines, patients were advised for ultrasonography of the hepatobiliary system every 6 months [27].

HCC was diagnosed by either histology or typical radiological features (arterial enhancement and venous wash-out by triphasic computed tomography [CT] scan or contrast magnetic resonance imaging [MRI]). Cirrhosis was diagnosed by one of the following 3 modalities: (1) imaging (USG/CT/MRI showing small liver, liver with an irregular outline, or features of portal hypertension including splenomegaly, varices and ascites), (2) transient elastography measured with a liver stiffness measurement of > 12 kilopascals [7], or (3) clinical features (thrombocytopenia, coagulopathy, ascites, hepatic hydrothorax, varices and hepatic encephalopathy).

### Diagnosis of significant fibrosis

The presence of significant fibrosis was defined by a Fibrosis 4 index (FIB-4) value of more than 1.45 [28]. At present, FIB-4 and aminostransferase-to-platelet ratio index (APRI) are the two widely studied non-invasive fibrosis markers based on readily available parameters (i.e. age, aminotransferase levels and platelet count). As a recent meta-analysis showed that FIB-4 outperformed APRI in detecting fibrosis, FIB-4 was used a surrogate marker for detecting significant fibrosis in our study [28].

### Treatment

The indications of commencing NA therapy included [1] a persistently elevated ALT level with high serum HBV DNA level (≥ 2,000 IU/mL), and/or [2] cirrhosis with detectable serum HBV DNA [29].

### Quantification of serum HBV DNA and WFA^+^-M2BP levels

Blood tests for HBV DNA and WFA^+^-M2BP levels were performed in serum stored at –20°C before treatment and during follow-up. Serum HBV DNA level was measured by the Cobas Taqman HBV Test (Roche Diagnostics, Branchburg, NJ, USA) with a lower limit of detection of 20 IU/mL. Serum WFA^+^-M2BP level was measured by HISCL M2BPGi reagent kit (Sysmex Corporation, Hyogo, Japan) on an automatic immunoanalyzer HISCL-800 (Sysmex Corporation, Hyogo, Japan) at Health-Tech Medical Laboratory Ltd in Hong Kong [8], and the measurement value was expressed as cutoff index (COI) by using the following equation:

Cutoff index (COI) = ([WFA^+^-M2BP]sample−[WFA^+^-M2BP]NC)/([WFA^+^-M2BP]PC−[WFA^+^-M2BP]NC). [WFA^+^-M2BP]sample refers to the WFA^+^-M2BP level of samples from recruited patients. NC and PC stand for negative control and positive control, respectively. The WFA^+^-M2BP level of PC from a calibration solution is standardized with a COI value of 1.0 [9].

### Statistical analyses

All statistical analyses were performed using R version 3.2.3 (R Foundation for Statistical Computing) statistical software. Continuous variables were expressed as median and interquartile range (IQR). Spearman's bivariate correlation was used to test the correlation between continuous variables. For comparisons of continuous variables between two groups, the Mann-Whitney *U*-test was used. For comparisons of categorical variables, the Chi-square test or Fisher's exact test were used when appropriate. The receiver operating curve was generated by plotting the ‘sensitivity’ against ‘1 - specificity’ at different values. The performance of WFA^+^-M2BP was measured in term of area under receiver operating curve (AUROC). By maximizing the Youden's index (i.e. sensitivity + specificity -1) from the AUROC analysis, optimal cutoff values for predicting HCC development were derived. In turn, the sensitivity, specificity, positive predictive value (PPV) and negative predictive value (NPV) were determined. The odds ratio (OR) of HCC development for different variables was derived from conditional logistic regression as the cases and controls were matched samples. Statistical significance was defined by a two-sided *p*-value of < 0.05.
